# Kine-Pox

**Published:** 1801-07

**Authors:** B. Waterhouse

**Affiliations:** Cambridge


### KINE-POX.

HAVING heard that ſeveral perſons, who had been inoculated for the *Kine-Pox* at Marblehead, have ſince taken the ſmall-pox, I wrote to the inoculator in that town, requeſting an accurate hiſtory of the buſineſs, and having waited a due time for an anſwer without receiving any, I can no longer defer giving you a few important facts reſpecting it. The public will then be better prepared to judge of the truth of my former aſſertion, namely, that "*such persons as have undergone the local disease and specific fever of the Kine-pox, are thereby rendered ever after unsusceptible of the Small-pox.*" For I have ſaid, in page 16 of my Pamphlet, that "the great queſtion which the profeſſional public were anxious to have reſolved, was, *whether a person who had been* FAIRLY *infected with the genuine* Cow, *or* Kine-pox, *was thereby secured against the* Small-pox?" The facts adduced in that Treatiſe ſatisfied the public. Let us now ſee if any have occurred ſince, ſufficiently ſtrong and clear to change their opinion.

I inoculated two, and but two inhabitants of Marblehead. One was a perſon whoſe name I have forgotten, who ſaid he would return to me in ſix days, that I might ſee if his inoculation had *taken*; but he returned no more. As I never ſaw him under the diſorder, I cannot be anſwerable for him. The other was the ſon of a practitioner, a boy of about eight or nine years of age. His father brought him to my houſe in Cambridge. I put the thread in his arm, and gave his father a ſmall portion of the ſame, but never ſaw nor heard any more of the boy afterwards, and of courſe I never knew whether he ever had the genuine *local affection* and *specific fever*, which conſtitute that peculiar diſtemper, which I have ſaid and ſtill believe ſecures the human ſyſtem from the Small-pox. Yet from from this boy's arm was taken, as I am informed, all the matter with which all the others in Marblehead were inoculated. If the matter therefore which I ufed did not give the genuine difeafe in the *firft* inftance, which happens very frequently, then all the cafes that followed it muft of courfe be *spurious*, and abfolutely incapable of fecuring the fyftem from an attack of the Small-pox. As this child's father had never feen the difeafe, it is no reflection on his fkill to fay that he was incapable of judging whether his fon, or his other patients, abfolutely had the genuine Kine-pox.—The wide avenue which thefe facts open to uncertainty muft be apparent to every difcerning individual. If the credit of the Kine-pox has been tried by the evidence from Marblehead only, thefe facts will have no fmall weight in pleading *an arreft of judgment*.

But Marblehead has not afforded the only inftance of a perfon having the Small after having been inoculated for the Kine-pox, and it is agreeable to the candor we profefs to bring forward all the facts that have come to our knowledge on this important fubject. Yefterday I received a letter from Hudfon, on the North-River. The writer fays, that in October laft he obtained fome Kine-pox matter from a gentleman who faid he had it of me, and with it he inoculated upwards of thirty people; of which number not above ten had the fymptomatic fever; of thefe ten five only had fymptoms with fome feverity; eight or ten more were fo flightly affected as to render it *doubtful* whether they had the proper fymptoms. The remainder were not in the *leaft* affected, except at the place where the infection was inferted. He then adds, that willing to fatisfy his own mind, and that of the inhabitants of the place, he determined to inoculate one of his patients with the Small-pox; and chofe for that purpofe one of the *doubtful clafs*, which patient broke out with the Small-pox the eleventh day after inoculation. This phyfician, who thinks like a man of fenfe, and writes like a gentleman, adds to the foregoing narrative the following reflections. "I am fenfible that I have committed an error. It would have been more prudent to have waited until further obfervation and information had inftructed me more in the nature of this difeafe. It, however," fays he, "excites my aftonifhment, that the fame matter fhould in one fubject produce the fpecific fever, and in another a painful tumor only, which maturates and difcharges freely without entering the fyftem. I beg leave, therefore, to enquire what are the certain criteria by which I may determine, in thofe cafes where the fymptoms are doubtful or obfcure, whether the fpecific change in the fyftem has taken place from the operation of the Kine-pox infection as will in future prevent the Small-pox?" At the fame time I received a letter from Tolland, in Connecticut, the writer equally unknown to me with that from Hudfon. The fubftance of it is, that a perfon came to his Small-pox Hofpital to be inoculated, after having been inoculated for the Kine-pox, and that he went regularly through the Small-pox. The account which the man gave of his Kine-pox inoculation, and its confequence, did not accord with the true difeafe. He faid he was inoculated by one Chandler, who told him that he had obtained his matter from me, and had been under my inftructions at Cambridge. This is not the only perfon who has impofed on the common country people by fimilar falfehoods.

The above inftances, in addition to what has occurred at Marblehead, ought to excite the ferious attention of all concerned in the Kine-pox inoculation. I expect to hear fimilar accounts from other quarters; for it is a fact, and a deplorable one, that a great many have been, and ftill are, inoculating with *spurious* matter, without knowing it, which matter produces a *spurious* difeafe, merely imitative of the *genuine* Kine-pox.

Apprehenfive of fome fuch accidents, I publifhed at a very early period of this bufinefs, that "there were fome circumftances, which, if not critically attended to, might bring the inoculation of this recently imported diftemper into a temporary difrepute. That Dr. Jenner, aware of fuch an accident, had pointed out the fallacious fources, whence a difeafe imitative of the Kine-pox may arife. The fources of this fpurious Kine-pox, as enumerated in my Treatife, page 26, are, Firft, That arifing from puftules on the nipples or udder of the cow, which puftules contain no fpecific virus. Secondly, From matter (although originally poffeffing the fpecific virus) which has fuffered a decompofition, either from putrefaction, or from any other caufe lefs obvious to the fenfes. Thirdly, From matter taken from an ulcer in an *advanced stage*, which ulcer arofe from a true Kine-pox. In the fame page I continue to fay, from Dr. Jenner, that when the inoculated part has degenerated into an ulcer, the matter, though it may poffefs the power of inflaming the patient's arm, is neverthelefs void of that fpecific virus requifite to produce the genuine difeafe, and, of courfe, INCAPABLE OF SECURING THE HUMAN SYSTEM AGAINST THE SMALL-POX.

And as if this was not fufficient warning, I quoted this paffage from Dr. Jenner. "I have often been foiled in my endeavours to communicate the Cow-pox by inoculation. An inflammation will fometimes fucceed the fcratch or puncture, and in a few days difappears without producing any further effect.

effect. Sometimes it will even produce an ichorous fluid, and yet the fyftem will not be affected."

And left the reader's attention fhould not after all this be roufed, I added, "Inftances of this kind are recorded to have happened in England, where the perfons were afterwards inoculated for the Small-pox, and TOOK THE DISORDER."

To all this, I now add an extract of a letter from Dr. Jenner to Dr. Pearfon, publifhed in the 1ft vol. of the Medical and Phyfical Journal, article the very firft; where, after fuggefting a caution refpecting the *state* of the matter employed in inoculating, which he thinks lofes its fpecific effect after it has loft its *limpid* quality, adds, "much precaution is therefore neceffary in the progrefs of the enquiry; and *this is my grand fear*, that the difcovery may fall into difcredit *from a want of that attention, in conducting the experiments which the subject requires*. For example, a perfon may conceive he has the Cow-pox matter on his lancet, when in fact, there may be only a little putrid pus; with this he inoculates, and excites a difeafe of fome kind, *but not such a one as will prevent the Small-pox*. Thus a delufive inference would be drawn, at once hurtful to the caufe, and particularly injurious to me. However, truth muft appear at laft."

If any practitioner engaged in the Kine-pox inoculation will not read what is written expreffly for information on this fubject, he muft be anfwerable for the confequences of his negligence. There are cafes, (efpecially in the medical department) where ignorance is converted into a crime.

Many have, to the idea of a diftemper, milder, fhorter, and fafer than the inoculated Small-pox, annexed the idea of lefs trouble in the communication and treatment of it. I hefitate not, on the contrary, to affirm, that putting the circumftances attending its contagion out of the queftion, there is no practitioner who has experienced both, but what will prefer inoculating for the Small-pox to that of the Kine-pox; the latter calling for much more attention, and exacting a much nicer difcernment than the former. Hence I will venture to predict, that the inoculation for the Kine-pox will not be a favourite branch of bufinefs with phyficians of extenfive practice in the larger fea-port towns.

Upon a deliberate review of the whole bufinefs, I have not feen, read, or heard, any thing that has any way diminifhed my intire confidence in the falutiferious confequences of the Kine-pox inoculation. I ftill continue firm in the opinion, that *those who have gone* FAIRLY *through this disorder can never have the Small-pox*.

Of

Of my fix children, the two eldeft had the Small-pox by inoculation in 1792; the other four were inoculated for the Kine-pox the paft fummer; and I deliberately declare, that I fhould feel as little reluctance in putting thefe four into a bed with a perfon under the confluent Small-pox, as I fhould the two eldeft. More cannot be faid to teftify *my* confidence in the practice. Should any one afk, if I would rifk fuch a trial with every one I inoculated? I anfwer, *No.* For about two or three weeks in the month of November the matter feemed to have undergone a *deterioration* in my hands. I inoculated feveral perfons four or five times over before I could communicate the difeafe. To three or four I never could communicate it. And fome ftill remain in that fhade of ambiguity, that I am ftill uncertain whether they have paffed *fairly* through the difeafe or not. Next Spring I hope to put thefe cafes beyond all doubt. As to thofe whom I have inoculated, and never feen afterwards, I do not confider myfelf as anfwerable for their fuccefs; for many came with an intention that I fhould never fee them again or even know their names.

I have entertained an opinion, which fome may think, perhaps, not ftrictly philofophical, namely, that *the Kine-pox matter becomes milder as it recedes from the cow*, and that in procefs of time it gets worn out, and, like the feed of fome vegetables, needs renewal. My own obfervations have inclined me to this opinion. When it is known that all the Vaccine matter hitherto ufed in America (fome received at Ipfwich excepted) came from *less than two inches* of infected thread which I received from Bath in England laft June, this idea will not appear altogether abfurd. It is certain, that when I firft began to inoculate, viz. in July laft, a very fmall portion of the thread never failed to communicate the difeafe on the very firft trial, when the patients followed their ordinary bufinefs and took no medicine. Whereas, at this time, I ufe fix times the quantity of infected thread, keep the patients within doors, give them fmall dofes of calomel and antimony, affifted by pediluvim at bed-time. It is not improbable that further experience may modify this opinion and practice.

Under an impreffion of the *deterioration* of the Vaccine matter, I have written to Drs. Jenner, Pearfon, Woodville, Thornton, Haygarth, Lettfom, and the Directors of the Vaccine Inftitution, and to feveral practitioners who have had the moft concern in this inoculation, and have fuggefted an arrangement by which I may expect a fupply of matter, as *direct from the cow* as poffible, every four weeks throughout the coming year, or until we get the Kine-pox naturalized in this country as much as it is in England. Previoufly to which, I

fhall

ſhall venture another communication, entitled "*Practical Obſervations on the local Appearance, Symptoms, and Mode of Treating the Kine-pox,*" which I hope to make through the medium of the Centinel and of the Mercury.

*Cambridge, Dec. 20, 1800.* B. WATERHOUSE.

---

### From the PRESIDENT of the UNITED STATES to Dr. WATERHOUSE.

SIR, *Washington, Dec.* 25, 1800.

I received laſt night, and have read with great ſatisfaction, your Pamphlet on the ſubject of the Kine-pox, and pray you to accept my thanks for the communication of it. I had before attended to your publications on the ſubject in the Newspapers, and took much intereſt in the reſult of the experiments you were making. Every friend of humanity muſt look with pleaſure on this diſcovery, by which one evil more is withdrawn from the condition of man; and muſt contemplate the poſſibility, that future improvements and diſcoveries may ſtill more and more leſſen the catalogue of evils. In this line of proceeding you deſerve well of your country; and I pray you, Sir, accept my portion of the tribute due to you, and aſſurances of the high conſideration and reſpect with which I am,

SIR,

Your moſt obedient humble ſervant,

(COPY.) THO. JEFFERSON.

*To Dr.* WATERHOUSE, *Cambridge.*

